# Supplementary material for: Radiogenomic biomarkers for immunotherapy in glioblastoma: A systematic review of magnetic resonance imaging studies
Source: Neurooncol Adv. 2024 Apr 5;6(1):vdae055. doi: 10.1093/noajnl/vdae055 (PMC11046988; doi:10.1093/noajnl/vdae055)
Supplement: vdae055_suppl_Supplementary_Tables_S1-S5_Figure_S1 [file vdae055_suppl_supplementary_tables_s1-s5_figure_s1.docx]

**Supplementary Material for**

**Radiogenomic biomarkers for immunotherapy in glioblastoma: a systematic review of magnetic resonance imaging studies**

**Ghimire Prajwal^1,2^, Kinnersley Ben^3^, Altmann Andre^3^, Golestan Karami^4^, Arumugam Prabhu^4^, Houlston Richard^5^, Ashkan Keyoumars^2^, Modat Marc^1^, Booth C Thomas^1,2^**

1. School of Biomedical Engineering & Imaging Sciences, King’s College London, UK

2. Kings College Hospital NHS Foundation Trust, London, UK

3. University College London, London, UK

4. Genomics England, London, UK

5. The Institute of Cancer Research, Sutton, UK

**Supplementary Table S1: Preferred Reporting Items for Systematic Reviews and Meta-Analyses (PRISMA) checklist**

**Supplementary Table S2: Search strategy for PubMed and OVID**

**Supplementary Table S3: CLAIM : Checklist for Artificial Intelligence in Medical Imaging**

**Supplementary Table S4: Imaging data collection, processing and harmonization techniques deployed in the studies**

**Supplementary Table S5: Genomic data collection, processing and harmonization techniques deployed in the studies**

**Supplementary Figure S1: QUADAS 2 assessment of risk of bias and applicability concerns**

**Supplementary Table S1**: Preferred Reporting Items for Systematic Reviews and Meta-Analyses (PRISMA) checklist^1^

| **Section and Topic** | **Item #** | **Checklist item** | **Location where item is reported** |
| --- | --- | --- | --- |
| **TITLE** | | |  |
| Title | 1 | Identify the report as a systematic review. | 1 |
| **ABSTRACT** | | |  |
| Abstract | 2 | See the PRISMA 2020 for Abstracts checklist. | 3,4 |
| **INTRODUCTION** | | |  |
| Rationale | 3 | Describe the rationale for the review in the context of existing knowledge. | 5 |
| Objectives | 4 | Provide an explicit statement of the objective(s) or question(s) the review addresses. | 5 |
| **METHODS** | | |  |
| Eligibility criteria | 5 | Specify the inclusion and exclusion criteria for the review and how studies were grouped for the syntheses. | 6 |
| Information sources | 6 | Specify all databases, registers, websites, organisations, reference lists and other sources searched or consulted to identify studies. Specify the date when each source was last searched or consulted. | 6 |
| Search strategy | 7 | Present the full search strategies for all databases, registers and websites, including any filters and limits used. | 6 |
| Selection process | 8 | Specify the methods used to decide whether a study met the inclusion criteria of the review, including how many reviewers screened each record and each report retrieved, whether they worked independently, and if applicable, details of automation tools used in the process. | 6 |
| Data collection process | 9 | Specify the methods used to collect data from reports, including how many reviewers collected data from each report, whether they worked independently, any processes for obtaining or confirming data from study investigators, and if applicable, details of automation tools used in the process. | 6,7 |
| Data items | 10a | List and define all outcomes for which data were sought. Specify whether all results that were compatible with each outcome domain in each study were sought (e.g. for all measures, time points, analyses), and if not, the methods used to decide which results to collect. | 6,7 |
|  | 10b | List and define all other variables for which data were sought (e.g. participant and intervention characteristics, funding sources). Describe any assumptions made about any missing or unclear information. | 6,7 |
| Study risk of bias assessment | 11 | Specify the methods used to assess risk of bias in the included studies, including details of the tool(s) used, how many reviewers assessed each study and whether they worked independently, and if applicable, details of automation tools used in the process. | 7 |
| Effect measures | 12 | Specify for each outcome the effect measure(s) (e.g. risk ratio, mean difference) used in the synthesis or presentation of results. | 6,7 |
| Synthesis methods | 13a | Describe the processes used to decide which studies were eligible for each synthesis (e.g. tabulating the study intervention characteristics and comparing against the planned groups for each synthesis (item #5)). | n/a |
|  | 13b | Describe any methods required to prepare the data for presentation or synthesis, such as handling of missing summary statistics, or data conversions. | 6,7 |
|  | 13c | Describe any methods used to tabulate or visually display results of individual studies and syntheses. | 6,7 |
|  | 13d | Describe any methods used to synthesize results and provide a rationale for the choice(s). If meta-analysis was performed, describe the model(s), method(s) to identify the presence and extent of statistical heterogeneity, and software package(s) used. | 6,7 |
|  | 13e | Describe any methods used to explore possible causes of heterogeneity among study results (e.g. subgroup analysis, meta-regression). | N/A |
|  | 13f | Describe any sensitivity analyses conducted to assess robustness of the synthesized results. | N/A |
| Reporting bias assessment | 14 | Describe any methods used to assess risk of bias due to missing results in a synthesis (arising from reporting biases). | 7 |
| Certainty assessment | 15 | Describe any methods used to assess certainty (or confidence) in the body of evidence for an outcome. | N/A |
| **RESULTS** | | |  |
| Study selection | 16a | Describe the results of the search and selection process, from the number of records identified in the search to the number of studies included in the review, ideally using a flow diagram. | 8-13 |
|  | 16b | Cite studies that might appear to meet the inclusion criteria, but which were excluded, and explain why they were excluded. | 8-13 |
| Study characteristics | 17 | Cite each included study and present its characteristics. | 8-13 |
| Risk of bias in studies | 18 | Present assessments of risk of bias for each included study. | 8-13 |
| Results of individual studies | 19 | For all outcomes, present, for each study: (a) summary statistics for each group (where appropriate) and (b) an effect estimate and its precision (e.g. confidence/credible interval), ideally using structured tables or plots. | 8-13 |
| Results of syntheses | 20a | For each synthesis, briefly summarise the characteristics and risk of bias among contributing studies. | 8-13 |
|  | 20b | Present results of all statistical syntheses conducted. If meta-analysis was done, present for each the summary estimate and its precision (e.g. confidence/credible interval) and measures of statistical heterogeneity. If comparing groups, describe the direction of the effect. | 8-13 |
|  | 20c | Present results of all investigations of possible causes of heterogeneity among study results. | 8-13 |
|  | 20d | Present results of all sensitivity analyses conducted to assess the robustness of the synthesized results. | N/A |
| Reporting biases | 21 | Present assessments of risk of bias due to missing results (arising from reporting biases) for each synthesis assessed. | 8-13 |
| Certainty of evidence | 22 | Present assessments of certainty (or confidence) in the body of evidence for each outcome assessed. | 8-13 |
| **DISCUSSION** | | |  |
| Discussion | 23a | Provide a general interpretation of the results in the context of other evidence. | 15- 19 |
|  | 23b | Discuss any limitations of the evidence included in the review. | 15-19 |
|  | 23c | Discuss any limitations of the review processes used. | 15-19 |
|  | 23d | Discuss implications of the results for practice, policy, and future research. | 15-19 |
| **OTHER INFORMATION** | | |  |
| Registration and protocol | 24a | Provide registration information for the review, including register name and registration number, or state that the review was not registered. | 3 |
|  | 24b | Indicate where the review protocol can be accessed, or state that a protocol was not prepared. | 3 |
|  | 24c | Describe and explain any amendments to information provided at registration or in the protocol. | N/A |
| Support | 25 | Describe sources of financial or non-financial support for the review, and the role of the funders or sponsors in the review. | 20 |
| Competing interests | 26 | Declare any competing interests of review authors. | 20 |
| Availability of data, code and other materials | 27 | Report which of the following are publicly available and where they can be found: template data collection forms; data extracted from included studies; data used for all analyses; analytic code; any other materials used in the review. | 20 |

**Supplementary Table S2: Search Strategy for PubMed and OVID**

| **Search databases** | **Search Terms** | **Translations/ Filters** |
| --- | --- | --- |
| **PubMed Search** | Search: **(Imaging or MRI) AND (high grade glioma OR Glioblastoma) AND (immunotherapy OR immune biomarkers)**  ("image"[All Fields] OR "image s"[All Fields] OR "imaged"[All Fields] OR "imager"[All Fields] OR "imager s"[All Fields] OR "imagers"[All Fields] OR "images"[All Fields] OR "imaging"[All Fields] OR "imaging s"[All Fields] OR "imagings"[All Fields] OR ("magnetic resonance imaging"[MeSH Terms] OR ("magnetic"[All Fields] AND "resonance"[All Fields] AND "imaging"[All Fields]) OR "magnetic resonance imaging"[All Fields] OR "mri"[All Fields])) AND ("glioma"[MeSH Terms] OR "glioma"[All Fields] OR ("high"[All Fields] AND "grade"[All Fields] AND "glioma"[All Fields]) OR "high grade glioma"[All Fields] OR ("glioblastoma"[MeSH Terms] OR "glioblastoma"[All Fields] OR "glioblastomas"[All Fields])) AND ("immunotherapy"[MeSH Terms] OR "immunotherapy"[All Fields] OR "immunotherapies"[All Fields] OR "immunotherapy s"[All Fields] OR (("immune"[All Fields] OR "immuned"[All Fields] OR "immunes"[All Fields] OR "immunisation"[All Fields] OR "vaccination"[MeSH Terms] OR "vaccination"[All Fields] OR "immunization"[All Fields] OR "immunization"[MeSH Terms] OR "immunisations"[All Fields] OR "immunizations"[All Fields] OR "immunise"[All Fields] OR "immunised"[All Fields] OR "immuniser"[All Fields] OR "immunisers"[All Fields] OR "immunising"[All Fields] OR "immunities"[All Fields] OR "immunity"[MeSH Terms] OR "immunity"[All Fields] OR "immunization s"[All Fields] OR "immunize"[All Fields] OR "immunized"[All Fields] OR "immunizer"[All Fields] OR "immunizers"[All Fields] OR "immunizes"[All Fields] OR "immunizing"[All Fields]) AND ("biomarker s"[All Fields] OR "biomarkers"[MeSH Terms] OR "biomarkers"[All Fields] OR "biomarker"[All Fields]))) | **Translations**  **Imaging:** "image"[All Fields] OR "image's"[All Fields] OR "imaged"[All Fields] OR "imager"[All Fields] OR "imager's"[All Fields] OR "imagers"[All Fields] OR "images"[All Fields] OR "imaging"[All Fields] OR "imaging's"[All Fields] OR "imagings"[All Fields]  **MRI:** "magnetic resonance imaging"[MeSH Terms] OR ("magnetic"[All Fields] AND "resonance"[All Fields] AND "imaging"[All Fields]) OR "magnetic resonance imaging"[All Fields] OR "mri"[All Fields]  **high grade glioma:** "glioma"[MeSH Terms] OR "glioma"[All Fields] OR ("high"[All Fields] AND "grade"[All Fields] AND "glioma"[All Fields]) OR "high grade glioma"[All Fields]  **Glioblastoma:** "glioblastoma"[MeSH Terms] OR "glioblastoma"[All Fields] OR "glioblastomas"[All Fields] OR "glioblastoma's"[All Fields]  **immunotherapy:** "immunotherapy"[MeSH Terms] OR "immunotherapy"[All Fields] OR "immunotherapies"[All Fields] OR "immunotherapy's"[All Fields]  **immune:** "immune"[All Fields] OR "immuned"[All Fields] OR "immunes"[All Fields] OR "immunisation"[All Fields] OR "vaccination"[MeSH Terms] OR "vaccination"[All Fields] OR "immunization"[All Fields] OR "immunization"[MeSH Terms] OR "immunisations"[All Fields] OR "immunizations"[All Fields] OR "immunise"[All Fields] OR "immunised"[All Fields] OR "immuniser"[All Fields] OR "immunisers"[All Fields] OR "immunising"[All Fields] OR "immunities"[All Fields] OR "immunity"[MeSH Terms] OR "immunity"[All Fields] OR "immunization's"[All Fields] OR "immunize"[All Fields] OR "immunized"[All Fields] OR "immunizer"[All Fields] OR "immunizers"[All Fields] OR "immunizes"[All Fields] OR "immunizing"[All Fields]  **biomarkers:** "biomarker's"[All Fields] OR "biomarkers"[MeSH Terms] OR "biomarkers"[All Fields] OR "biomarker"[All Fields] |
| **OVID** | 1 glioblastoma.mp. [mp=ti, ab, hw, tn, ot, dm, mf, dv, kf, fx, dq, bt, nm, ox, px, rx, ui, sy] 140295  2 high grade glioma.mp. [mp=ti, ab, hw, tn, ot, dm, mf, dv, kf, fx, dq, bt, nm, ox, px, rx, ui, sy] 10234  3 1 or 2 146376  4 immunotherapy.mp. [mp=ti, ab, hw, tn, ot, dm, mf, dv, kf, fx, dq, bt, nm, ox, px, rx, ui, sy] 395375  5 immune cell markers.mp. [mp=ti, ab, hw, tn, ot, dm, mf, dv, kf, fx, dq, bt, nm, ox, px, rx, ui, sy] 730  6 4 or 5 395998  7 MRI.mp. [mp=ti, ab, hw, tn, ot, dm, mf, dv, kf, fx, dq, bt, nm, ox, px, rx, ui, sy] 820286  8 imaging.mp. [mp=ti, ab, hw, tn, ot, dm, mf, dv, kf, fx, dq, bt, nm, ox, px, rx, ui, sy] 4515563  9 7 or 8 4581677  10 3 and 6 and 9 960 | **Filter**: conference papers; non-glioma; paediatrics, vaccine trials; non-human; non glioma; non-imaging; Vaccine treatment; encephalitis; invasive studies including intratumoral injections, nanoparticles injection; **Included**: English, abstracts, humans, adults |

**Supplementary Table S3:** CLAIM: Checklist for Artificial Intelligence in Medical Imaging^2^

| Section / Topic | No. | Item |  |
| --- | --- | --- | --- |
| TITLE / ABSTRACT |  |  |  |
|  | **1** | Identification as a study of AI methodology, specifying the category of technology used (e.g., deep learning) | **√** |
|  | **2** | Structured summary of study design, methods, results, and conclusions | **√** |
| INTRODUCTION |  |  |  |
|  | **3** | Scientific and clinical background, including the intended use and clinical role of the AI approach | **√** |
|  | **4** | Study objectives and hypotheses | **√** |
| METHODS |  |  |  |
| *Study Design* | **5** | Prospective or retrospective study | **√** |
|  | **6** | Study goal, such as model creation, exploratory study, feasibility study, non-inferiority trial | **√** |
| *Data* | **7** | Data sources | **√** |
|  | **8** | Eligibility criteria: how, where, and when potentially eligible participants or studies were identified (e.g., symptoms, results from previous tests, inclusion in registry, patient-care setting, location, dates) | **√** |
|  | **9** | Data pre-processing steps | **√** |
|  | **10** | Selection of data subsets, if applicable | **√** |
|  | **11** | Definitions of data elements, with references to Common Data Elements | **√** |
|  | **12** | De-identification methods | **N/A** |
|  | **13** | How missing data were handled | **N/A** |
| *Ground Truth* | **14** | Definition of ground truth reference standard, in sufficient detail to allow replication | **√** |
|  | **15** | Rationale for choosing the reference standard (if alternatives exist) | **√** |
|  | **16** | Source of ground-truth annotations; qualifications and preparation of annotators | **N/A** |
|  | **17** | Annotation tools | **N/A** |
|  | **18** | Measurement of inter- and intrarater variability; methods to mitigate variability and/or resolve discrepancies | **√** |
| *Data Partitions* | **19** | Intended sample size and how it was determined | **√** |
|  | **20** | How data were assigned to partitions; specify proportions | **N/A** |
|  | **21** | Level at which partitions are disjoint (e.g., image, study, patient, institution) | **√** |
| *Model* | **22** | Detailed description of model, including inputs, outputs, all intermediate layers and connections | **√** |
|  | **23** | Software libraries, frameworks, and packages | **√** |
|  | **24** | Initialization of model parameters (e.g., randomization, transfer learning) | **√** |
| *Training* | **25** | Details of training approach, including data augmentation, hyperparameters, number of models trained | **√** |
|  | **26** | Method of selecting the final model | **√** |
|  | **27** | Ensembling techniques, if applicable | **N/A** |
| *Evaluation* | **28** | Metrics of model performance | **√** |
|  | **29** | Statistical measures of significance and uncertainty (e.g., confidence intervals) | **√** |
|  | **30** | Robustness or sensitivity analysis | **√** |
|  | **31** | Methods for explainability or interpretability (e.g., saliency maps), and how they were validated | **√** |
|  | **32** | Validation or testing on external data | **√** |
| RESULTS |  |  |  |
| *Data* | **33** | Flow of participants or cases, using a diagram to indicate inclusion and exclusion | **√** |
|  | **34** | Demographic and clinical characteristics of cases in each partition | **√** |
| *Model performance* | **35** | Performance metrics for optimal model(s) on all data partitions | **√** |
|  | **36** | Estimates of diagnostic accuracy and their precision (such as 95% confidence intervals) | **√** |
|  | **37** | Failure analysis of incorrectly classified cases | **N/A** |
| DISCUSSION |  |  |  |
|  | **38** | Study limitations, including potential bias, statistical uncertainty, and generalizability | **√** |
|  | **39** | Implications for practice, including the intended use and/or clinical role | **√** |
| OTHER INFORMATION |  |  |  |
|  | **40** | Registration number and name of registry | **√** |
|  | **41** | Where the full study protocol can be accessed | **√** |
|  | **42** | Sources of funding and other support; role of funders | **√** |

**Supplemental Table S4: Imaging data Collection, processing and harmonization techniques deployed in the studies**

| **Paper** | **Tesla Strength** | **MRI Manufacturer** | **MRI Sequences** | **Segmentation of VOI (Tool used)** | **Image processing** | **Harmonization** |
| --- | --- | --- | --- | --- | --- | --- |
| Cho et al^23^ | 3T | Siemens* | Pre-operative T2 FLAIR, T1 CE, ADC, DSC | Manual  (Polygonal ROI) | Co-registration of structural (FLAIR, T1 CE) images and the nCBV, ADC maps was performed using nordicICE* software  ADC maps calculated on a voxel-by-voxel basis with software incorporated into the local MRI unit.  Relative CBV was obtained with nordicICE* software. | N/A |
| Liao et al^24^ | N/A | N/A | Pre-operative T2 FLAIR | Manual  (3D slicer*) | ROI Feature extraction using Pyradiomics*.  Feature selection using importance index >0.04. | N/A |
| Jajamovich et al^26^ | 1.5T; 3T | GE*, Siemens* | Pre-operative T1, T1 CE, DWI, ADC | Semi-automatic (GrowCut*) | Co-registration of all scans to T1 CE (MATLAB*).  T1 and T1 CE images used to compute relative enhancement; followed by segmentation of enhancing region.  DWI images used to compute ADC maps;  b-values for ADC map obtained for each patient.  ADC histogram obtained from segmented VOI and ADC maps (MATLAB*). | N/A |
| Liu et al^27^ | 1.5T; 3T | GE*; Siemens* | Pre-operative  T2, T1 CE | Manual  (ITK-SNAP*) | Resampling(1*1*1).  Registration using SPM12 tool (MATLAB*).  Skull stripping using BET*.  N4 bias field correction.  White stripe correction using WhiteStripe algorithm (R*).  Low-level non-linear filtering.  ROI radiomics extracted using Pyradiomics*. | N/A |
| Rao et al^28^ | N/A | N/A | Pre-operative T2, T2 FLAIR, T1, T1 CE | Not performed | Pre-extracted standardized VASARI features. | N/A |
| Narang et al^29^ | 1.5T; 3T | GE* | Pre-operative T2 FLAIR, T1 CE | Semi-automatic  (MITK 3M3*) | Non-parametric intensity non-uniformity normalization (N3) correction using Medical Image Processing Analysis and Visualization* software (v 7.2.0).  ROI features extracted using MATLAB* radiomics toolbox.  Feature selection using R* Boruta package. | N/A |
| Hsu et al^30^ | N/A | N/A | Pre-operative T1 CE, DWI | Manual  (N/A) | Local post processing protocol ; adjustment of image resolution to voxel size of 0.75 × 0.75 × 3.00 mm^3^.  Intensity normalization.  ADC maps registered to T1C images by a six-parameter rigid body transformation and mutual information algorithm.  Radiomic features extracted from T1C and ADC maps.  Feature selection based on correlation, importance, and information gain functions (R software*). | N/A |
| George et al^22^ | 1.5T, 3T | N/A | Pre-operative and 8-week post operative T2, T2 FLAIR, T1, T1 CE | Manual  (3D Slicer*) | Skull stripping (MATLAB*).  Correction of image intensity with N4 bias field correction.  Image intensities normalized with median and IQR of normal VOI intensity.  Resampled to 1mm and spatially registered to T1CE images (MATLAB*).  Radiomics feature extraction (MATLAB*).  Feature selection using variability and redundancy. | N/A |
| Qin et al^25^ | 1.5T, 3T | GE, Siemens | Pre-operative  T2 FLAIR, T1, T1 CE, ADC | Manual | ADC maps calculated using manufacturer supplied software.  Scans co-registered and resampled bilinearly to baseline T1CE (Mirada* software).  Registered (rigid/deformable).  Correctness of registration by visual inspection.  RANO measurements on T1 CE images; volumetric change on T2 FLAIR, T1 CE, ADC maps. | N/A |

**Siemens = Siemens Healthineers Erlangen, Germany; GE = General Electric Healthcare, Chicago, US; 3D Slicer = 3D Slicer open-source software, https://github.com/Slicer/Slicer; nordicICE = nordicICE software from Nordic Imaging Lab, Bergen, Norway; MATLAB = MATLAB, MathWorks, California, US; GrowCut = GrowCut algorithm, Moscow, Russia; ITK-SNAP = ITK-SNAP open-source software, http://www.itksnap.org/pmwiki/pmwiki.php; Pyradiomics = Pyradiomics open source algorithm, Havard, US; MITK 3M3 = The Medical Imaging Interaction Toolkit (MITK), open-source software, https://github.com/MITK/MITK; Mirada: Mirada software, Denver, US; R = R software, free statistical software for computing and graphics, https://www.r-project.org/; BET= Brain extraction tool, FMRIB software library, Oxford, UK*

*ADC: apparent diffusion coefficient; CE: contrast enhanced; T: tesla, PCR: polymerase chain reaction; T: tesla; N/A: not available; VASARI: Visually AcceSAble Rembrandt Images; ADC: apparent diffusion coefficient; DWI: diffusion weighted imaging; FLAIR: fluid attenuated inversion recovery.*

**Supplementary Table S5: Genomic data collection, processing and harmonization techniques deployed in the studies**

| **Paper** | **Genomic data extraction technique** | **Normalization method** | **Processing / harmonization** |
| --- | --- | --- | --- |
| Cho et al^23^ | PCR for RNA isolation | RNA expression level normalized by using the highest expressed immune cell marker of each patient. | N/A |
| Liao et al^24^ | RNA sequencing related gene expression levels | N/A | N/A |
| Jajamovich et al^26^ | RNA sequencing related gene expression levels | Affymetrix level 3 mRNA data.  Filtered genes were normalized using Z score. | Genes filtered using observed variance and entropy across samples.  Genes were ranked according to the variance and lower 40^th^ percentile were discarded.  Genes with entropy in the lower 40^th^ percentile were discarded. |
| Liu et al^27^ | RNA sequencing related gene expression levels | Fpkm values were converted to TPM values. | Combat for batch harmonization (R*: sva package). |
| Rao et al^28^ | RNA sequencing related gene expression levels  miRNA expression data | Affymetrix level 3 mRNA and miRNA data. | N/A |
| Narang et al^29^ | RNA sequencing related gene expression levels  Immunohistochemistry based CD3 counts | Z scores were used to normalized mRNA expression values and immunohistochemistry based CD3 counts. | N/A |
| Hsu et al^30^ | RNA sequencing related gene expression levels  RNA microarray related expression levels | Level 1 RNA sequencing data were converted to fpkm values after processing with HISAT2* and STRINGTIE*.  Microarray data normalized using locally weighted scatterplot smoothing (LOWESS)* normalization method. | N/A |
| George et al^22^ | Not applicable as post hoc analysis of Phase II immunotherapy clinical trial data; no genomic data extracted | N/A | N/A |
| Qin et al^25^ | Not applicable as analysis of immunotherapy clinical trial data; no genomic data extracted | N/A | N/A |

** R = R software, free statistical software for computing and graphics, https://www.r-project.org/; HISAT2 and STRINGTIE = command-line tools based on C++ language for processing RNA sequencing data; LOWESS = normalisation method, can be implemented in R or python or command line; Affymetrix = Affymetrix microarrays, Thermo Fisher Scientific, Massachusetts, US*

*N/A: not available; PCR: polymerase chain reaction; RNA: ribonucleic acid, fpkm: fragments per kilobase per million; tpm: transcripts per kilobase million; sva: surrogate variable analysis; miRNA: microRNA; mRNA: messenger RNA*

**Supplementary Figure S1:** QUADAS 2 assessment of risk of bias and applicability concerns^3^


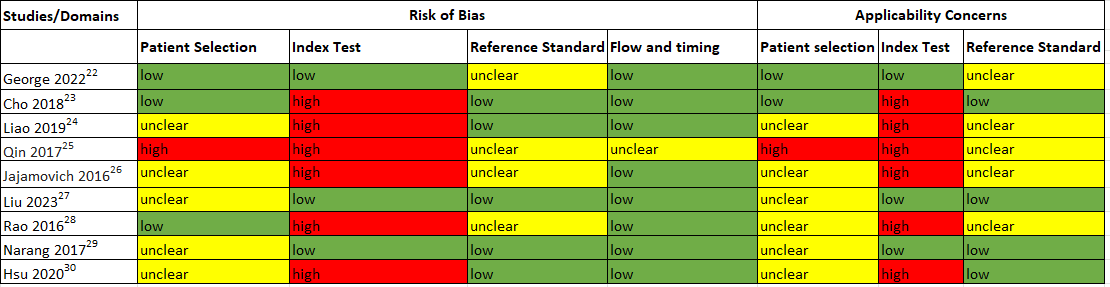


**References:**

1. Page MJ, McKenzie JE, Bossuyt PM, Boutron I, Hoffmann TC, Mulrow CD, et al. The PRISMA 2020 statement: an updated guideline for reporting systematic reviews. BMJ 2021;372:n71. doi: 10.1136/bmj.n71
2. Mongan J, Moy L, Kahn CE Jr. Checklist for Artificial Intelligence in Medical Imaging (CLAIM): a guide for authors and reviewers. Radiol Artif Intell 2020; 2(2):e200029. https://doi.org/10.1148/ryai.2020200029
3. Whiting PF, Rutjes AW, Westwood ME, et al. QUADAS-2: a revised tool for the quality assessment of diagnostic accuracy studies. Ann Intern Med. 2011;155(8):529–536.
